# 4-{4-Methyl-2-[(meth­yl)(2-methyl­phen­yl)amino]-1,3-thia­zol-5-yl}-*N*-(3-methyl­phen­yl)pyrimidin-2-amine

**DOI:** 10.1107/S1600536810053560

**Published:** 2011-01-08

**Authors:** Hai-Bo Shi, Feng Xu, Hai-Bo Li, Wei-Xiao Hu

**Affiliations:** a Zhejiang Pharmaceutical College, Ningbo 315100, People’s Republic of China; b College of Pharmaceutical Science, Zhejiang University of Technology, Hangzhou 310032, People’s Republic of China; c Taizhou Vocational & Technical College, Taizhou 318000, People’s Republic of China; d Nantong Center for Disease Control and Prevention, Nantong 226007, People’s Republic of China

## Abstract

In the title compound, C_23_H_23_N_5_S, the thia­zole ring and pyrimidine ring are almost coplanar, making a dihedral angle of 4.02 (9)°. in the crystal, weak inter­molecular N—H⋯N inter­actions link pairs of molecules into centrosymmetric dimers.

## Related literature

For general background to the biological activity of thia­zole derivatives, see: Narayana *et al.* (2004 ▶). For the synthesis of the title compound, see: Bredereck *et al.* (1964 ▶). 
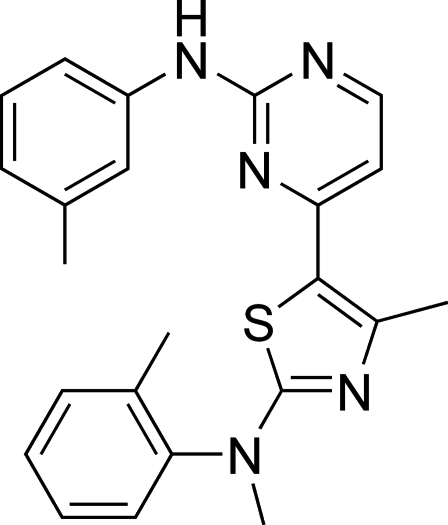


## Experimental

### 

#### Crystal data

C_23_H_23_N_5_S
*M* *_r_* = 401.52
Triclinic, 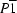

*a* = 7.886 (2) Å
*b* = 9.576 (3) Å
*c* = 13.531 (4) Å
α = 86.590 (9)°
β = 81.657 (7)°
γ = 85.926 (8)°
*V* = 1007.2 (5) Å^3^
*Z* = 2
Mo *K*α radiation
μ = 0.18 mm^−1^
*T* = 103 K
0.53 × 0.37 × 0.15 mm

#### Data collection

Rigaku FC10/Saturn724+ diffractometer
Absorption correction: multi-scan (*CrystalClear*; Rigaku/MSC, 2008 ▶) *T*_min_ = 0.910, *T*_max_ = 0.973
9716 measured reflections
4535 independent reflections
3652 reflections with *I* > 2σ(*I*)
*R*_int_ = 0.025

#### Refinement

*R*[*F* ^2^ > 2σ(*F* ^2^)] = 0.053
*wR*(*F* ^2^) = 0.145
*S* = 1.04
4535 reflections
267 parameters
H-atom parameters constrained
Δρ_max_ = 0.88 e Å^−3^
Δρ_min_ = −0.29 e Å^−3^

### 

Data collection: *CrystalClear* (Rigaku/MSC, 2008 ▶); cell refinement: *CrystalClear*; data reduction: *CrystalClear*; program(s) used to solve structure: *SHELXS97* (Sheldrick, 2008 ▶); program(s) used to refine structure: *SHELXL97* (Sheldrick, 2008 ▶); molecular graphics: *SHELXTL* (Sheldrick, 2008 ▶); software used to prepare material for publication: *publCIF* (Westrip, 2010 ▶).

## Supplementary Material

Crystal structure: contains datablocks I, global. DOI: 10.1107/S1600536810053560/ng5078sup1.cif

Structure factors: contains datablocks I. DOI: 10.1107/S1600536810053560/ng5078Isup2.hkl

Additional supplementary materials:  crystallographic information; 3D view; checkCIF report

## Figures and Tables

**Table 1:** Hydrogen-bond geometry (Å, °)

*D*—H⋯*A*	*D*—H	H⋯*A*	*D*⋯*A*	*D*—H⋯*A*
N5—H5*N*⋯N3^i^	0.88	2.22	3.097 (2)	173

Symmetry code: (i) 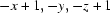.

**Fig. 1.**
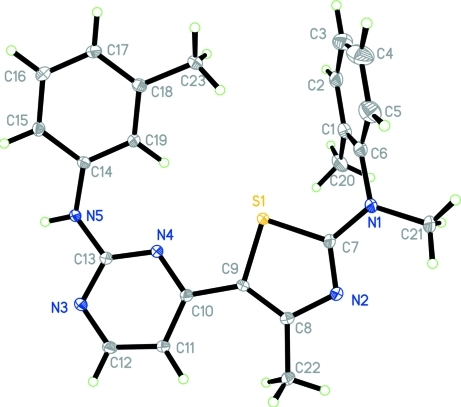
The structure of (I), shown with 30% probability displacement ellipsoids.

**Fig. 2.**
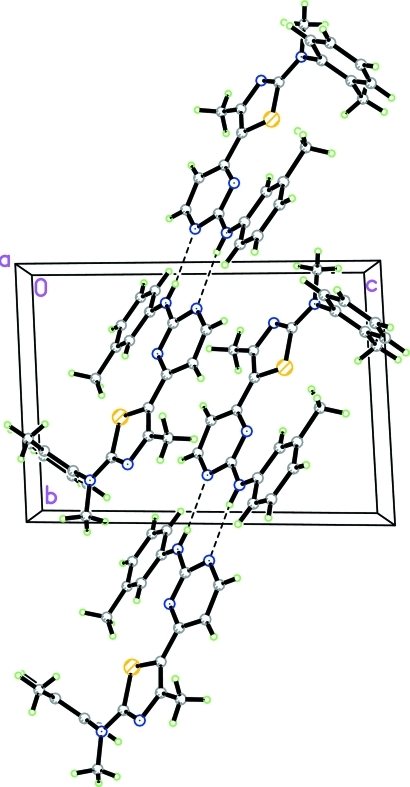
Packing of the molecules down a axis. Dashed lines denote intermolecular N—H···N hydrogen bonds.

## supplementary crystallographic information

### Comment

Thiazole derivatives are found to be associated with various biological activities (Narayana *et al.*, 2004). In order to further study the structure-activity relationship (SAR) of the thiazolyl-pyrimidine derivatives, we introduced arylamino group into 2-position of thiazole ring of thiazolyl-pyrimidine according to the general pyrimidine condensation method of Bredereck (Bredereck *et al.*, 1964). But, it was found that the obtained compound was not desired compound that confirmed by ^1^H NMR, MS. So, the structure of (I) was further determined using single-crystal X-ray diffraction.

The molecular structure of (I) is illustrated in Fig. 1. The thiazole ring (S1/C7/N2/C8/C9) and the pyrimidine ring (C10/C11/C12/N3/C13/N4) are almost planar, with a dihedral angle of 4.02 (9)°. The aniline rings (C1/C2/C3/C4/C5/C6/N1) and (C14/C15/C16/C17/C18/C19/N5) make dihedral angles of 80.96 (11) Å and 14.15 (9) Å with the thiazole ring, respectively. In the thiazole ring, the bond lengths S1—C7 [1.739 (2) Å], S1—C9 [1.748 (18) Å] and N2—C8 [1.372 (2) Å] correspond to typical single bond, and the C7—N2 [1.312 (2) Å], C8—C9 [1.373 (3) Å] belong to typical for double bonds. The crystal structure is stabilized by intermolecular weak N—H···N interactions (Fig. 2). Furthermore, every two molecules containing two N—H···N hydrogen bondings consists a dimer as octagon.

### Experimental

A mixture of 3-dimethylamino-1-[4-methyl-2-(methyl-*o*-tolyl-amino)-thiazol-5-yl]-propenone (1.575 g, 5 mmol) and NaOH (0.2 g, 5 mmol) in 2-methoxylethanol (20 ml) was treated with *N*-*m*-tolyl-guanidine carbonate (1.58 g, 7.5 mmol). The reaction mixture was heated at 383 K under N_2_ for 11 h. After concentration, the residue was filtered and washed liberally with ethanol and water. Recrystallization from acetone affored the title compound as dark yellow crystals, 1.17 g, m.p.455–458 K, yield 58.5%. Since the crystal product was not found to be suitable for X-ray diffraction studies,a few crystals were dissolved in 2-butanone, which was allowed to evaporate slowly to give yellow crystals of (I) suitable for X-ray diffraction studies. ^1^H NMR(CDCl_3_, TMS, 400 MHz, δp.p.m.): 8.24 (d, 1H, *J* = 5.2 Hz, py—H), 7.65 (s, 1H, Ar—H), 7.36–7.27 (m, 4H, Ar—H), 7.13–7.09 (m, 2H, Ar—H), 6.92 (s, 1H, Ar—H), 6.79 (d, 1H, *J* = 5.6 Hz, py—H), 3.49 (s, 3H, CH_3_), 2.61 (s, 3H, CH_3_), 2.29 (s, 3H, CH_3_), 2.15 (s, 3H, CH_3_). EIMS m/*z* (%): 401 (*M*^+^, 100), 386 (28), 368 (17), 283 (9), 222 (8), 129 (10), 118 (8), 98 (11), 91 (15), 83 (12), 73 (21), 65 (10), 57 (28).

### Refinement

All H atoms were placed in calculated positions (C—H 0.95–0.98 Å and N—H 0.87–0.89 Å) and refined as riding with *U*_iso_(H) = 1.2–1.22Ueq of the parent atom.

### Crystal data

**Table:**

C_23_H_23_N_5_S	*Z* = 2
*M**_r_* = 401.52	*F*(000) = 424
Triclinic, *P*1	*D*_x_ = 1.324 Mg m^−^^3^
Hall symbol: -P 1	Melting point = 455–458 K
*a* = 7.886 (2) Å	Mo *K*α radiation, λ = 0.71073 Å
*b* = 9.576 (3) Å	Cell parameters from 2962 reflections
*c* = 13.531 (4) Å	θ = 3.1–27.5°
α = 86.590 (9)°	µ = 0.18 mm^−^^1^
β = 81.657 (7)°	*T* = 103 K
γ = 85.926 (8)°	Chunk, yellow
*V* = 1007.2 (5) Å^3^	0.53 × 0.37 × 0.15 mm

### Data collection

**Table:**

Rigaku FC10/Saturn724+ diffractometer	4535 independent reflections
Radiation source: fine-focus sealed tube	3652 reflections with *I* > 2σ(*I*)
graphite	*R*_int_ = 0.025
Detector resolution: 28.5714 pixels mm^-1^	θ_max_ = 27.5°, θ_min_ = 3.2°
phi and ω scans	*h* = −9→10
Absorption correction: multi-scan (*CrystalClear*; Rigaku/MSC, 2008)	*k* = −12→12
*T*_min_ = 0.910, *T*_max_ = 0.973	*l* = −17→17
9716 measured reflections	

### Refinement

**Table:**

Refinement on *F*^2^	Primary atom site location: structure-invariant direct methods
Least-squares matrix: full	Secondary atom site location: difference Fourier map
*R*[*F*^2^ > 2σ(*F*^2^)] = 0.053	Hydrogen site location: inferred from neighbouring sites
*wR*(*F*^2^) = 0.145	H-atom parameters constrained
*S* = 1.04	*w* = 1/[σ^2^(*F*_o_^2^) + (0.0846*P*)^2^ + 0.3916*P*] where *P* = (*F*_o_^2^ + 2*F*_c_^2^)/3
4535 reflections	(Δ/σ)_max_ = 0.028
267 parameters	Δρ_max_ = 0.88 e Å^−^^3^
0 restraints	Δρ_min_ = −0.29 e Å^−^^3^

### Special details

**Table:**

Geometry. All e.s.d.'s (except the e.s.d. in the dihedral angle between two l.s. planes) are estimated using the full covariance matrix. The cell e.s.d.'s are taken into account individually in the estimation of e.s.d.'s in distances, angles and torsion angles; correlations between e.s.d.'s in cell parameters are only used when they are defined by crystal symmetry. An approximate (isotropic) treatment of cell e.s.d.'s is used for estimating e.s.d.'s involving l.s. planes.
Refinement. Refinement of *F*^2^ against ALL reflections. The weighted *R*-factor *wR* and goodness of fit *S* are based on *F*^2^, conventional *R*-factors *R* are based on *F*, with *F* set to zero for negative *F*^2^. The threshold expression of *F*^2^ > σ(*F*^2^) is used only for calculating *R*-factors(gt) *etc*. and is not relevant to the choice of reflections for refinement. *R*-factors based on *F*^2^ are statistically about twice as large as those based on *F*, and *R*- factors based on ALL data will be even larger.

### Fractional atomic coordinates and isotropic or equivalent isotropic displacement parameters (Å^2^)

**Table:**

	*x*	*y*	*z*	*U*_iso_*/*U*_eq_	
S1	0.49286 (6)	0.59975 (5)	0.26125 (4)	0.02216 (15)	
N1	0.5218 (2)	0.85391 (18)	0.16596 (14)	0.0291 (4)	
N2	0.7115 (2)	0.78346 (17)	0.28067 (13)	0.0240 (4)	
N3	0.5942 (2)	0.16585 (16)	0.49627 (12)	0.0200 (3)	
N4	0.5120 (2)	0.34459 (16)	0.37770 (12)	0.0187 (3)	
C1	0.4171 (3)	0.7392 (2)	0.03246 (17)	0.0313 (5)	
C2	0.2762 (3)	0.7123 (2)	−0.01462 (18)	0.0389 (6)	
H2	0.2943	0.6545	−0.0704	0.047*	
C3	0.1159 (4)	0.7658 (3)	0.0169 (2)	0.0467 (7)	
H3	0.0237	0.7459	−0.0171	0.056*	
C4	0.0846 (4)	0.8502 (4)	0.0990 (2)	0.0548 (8)	
H4	−0.0285	0.8877	0.1209	0.066*	
C5	0.2161 (4)	0.8783 (3)	0.14753 (19)	0.0430 (6)	
H5	0.1960	0.9358	0.2034	0.052*	
C6	0.3856 (3)	0.8205 (2)	0.11364 (17)	0.0313 (5)	
C7	0.5837 (3)	0.7599 (2)	0.23286 (15)	0.0224 (4)	
C8	0.7434 (3)	0.6704 (2)	0.34395 (15)	0.0214 (4)	
C9	0.6390 (2)	0.56111 (19)	0.34608 (14)	0.0189 (4)	
C10	0.6291 (2)	0.42802 (19)	0.40239 (14)	0.0179 (4)	
C11	0.7270 (3)	0.3854 (2)	0.47785 (14)	0.0217 (4)	
H11	0.8053	0.4448	0.4987	0.026*	
C12	0.7046 (3)	0.2525 (2)	0.52081 (15)	0.0225 (4)	
H12	0.7723	0.2206	0.5713	0.027*	
C13	0.4981 (2)	0.22012 (19)	0.42660 (14)	0.0187 (4)	
C14	0.2321 (2)	0.16692 (19)	0.35740 (14)	0.0189 (4)	
C15	0.1008 (2)	0.0738 (2)	0.37477 (14)	0.0206 (4)	
H15	0.1113	−0.0060	0.4192	0.025*	
C16	−0.0438 (3)	0.0964 (2)	0.32809 (15)	0.0232 (4)	
H16	−0.1304	0.0310	0.3394	0.028*	
C17	−0.0636 (3)	0.2142 (2)	0.26472 (15)	0.0224 (4)	
H17	−0.1636	0.2293	0.2330	0.027*	
C18	0.0630 (3)	0.3100 (2)	0.24782 (14)	0.0208 (4)	
C19	0.2116 (3)	0.2851 (2)	0.29292 (14)	0.0212 (4)	
H19	0.2997	0.3491	0.2797	0.025*	
C20	0.5933 (3)	0.6860 (3)	−0.0052 (2)	0.0422 (6)	
H20A	0.6656	0.7651	−0.0242	0.063*	
H20B	0.5914	0.6309	−0.0637	0.063*	
H20C	0.6400	0.6267	0.0473	0.063*	
C21	0.5830 (3)	0.9947 (2)	0.15159 (19)	0.0368 (6)	
H21A	0.6440	1.0133	0.2071	0.044*	
H21B	0.4851	1.0633	0.1495	0.044*	
H21C	0.6612	1.0023	0.0885	0.044*	
C22	0.8931 (3)	0.6763 (2)	0.40003 (19)	0.0328 (5)	
H22A	0.9559	0.7594	0.3763	0.039*	
H22B	0.9697	0.5919	0.3888	0.039*	
H22C	0.8516	0.6816	0.4717	0.039*	
C23	0.0426 (3)	0.4413 (2)	0.18208 (16)	0.0257 (4)	
H23A	0.1144	0.5124	0.2003	0.031*	
H23B	−0.0780	0.4770	0.1913	0.031*	
H23C	0.0781	0.4197	0.1119	0.031*	
N5	0.3751 (2)	0.13476 (17)	0.40750 (13)	0.0220 (4)	
H5N	0.3877	0.0470	0.4300	0.054 (9)*	

### Atomic displacement parameters (Å^2^)

**Table:**

	*U*^11^	*U*^22^	*U*^33^	*U*^12^	*U*^13^	*U*^23^
S1	0.0269 (3)	0.0208 (3)	0.0217 (3)	−0.00942 (19)	−0.01263 (19)	0.00710 (18)
N1	0.0400 (11)	0.0213 (8)	0.0299 (10)	−0.0104 (8)	−0.0187 (8)	0.0105 (7)
N2	0.0274 (9)	0.0211 (8)	0.0258 (9)	−0.0085 (7)	−0.0101 (7)	0.0045 (7)
N3	0.0207 (8)	0.0191 (8)	0.0211 (8)	−0.0012 (6)	−0.0070 (6)	0.0029 (6)
N4	0.0191 (8)	0.0181 (8)	0.0196 (8)	−0.0038 (6)	−0.0052 (6)	0.0015 (6)
C1	0.0364 (12)	0.0273 (11)	0.0297 (11)	−0.0028 (10)	−0.0069 (9)	0.0078 (9)
C2	0.0550 (16)	0.0320 (12)	0.0347 (13)	−0.0160 (11)	−0.0228 (11)	0.0133 (10)
C3	0.0469 (16)	0.0523 (16)	0.0453 (15)	−0.0117 (13)	−0.0219 (12)	0.0092 (12)
C4	0.0339 (14)	0.077 (2)	0.0510 (17)	0.0081 (14)	−0.0049 (12)	0.0043 (15)
C5	0.0512 (16)	0.0468 (15)	0.0304 (12)	0.0032 (12)	−0.0068 (11)	−0.0027 (11)
C6	0.0407 (13)	0.0279 (11)	0.0280 (11)	−0.0113 (10)	−0.0151 (9)	0.0115 (9)
C7	0.0269 (10)	0.0197 (9)	0.0218 (10)	−0.0073 (8)	−0.0070 (8)	0.0047 (7)
C8	0.0203 (9)	0.0218 (9)	0.0235 (10)	−0.0040 (8)	−0.0071 (7)	0.0000 (8)
C9	0.0202 (9)	0.0191 (9)	0.0186 (9)	−0.0026 (7)	−0.0076 (7)	0.0024 (7)
C10	0.0171 (9)	0.0173 (9)	0.0198 (9)	−0.0013 (7)	−0.0041 (7)	−0.0005 (7)
C11	0.0221 (10)	0.0215 (9)	0.0237 (10)	−0.0047 (8)	−0.0097 (8)	0.0017 (8)
C12	0.0240 (10)	0.0217 (9)	0.0235 (10)	−0.0004 (8)	−0.0104 (8)	0.0000 (8)
C13	0.0184 (9)	0.0191 (9)	0.0189 (9)	−0.0018 (7)	−0.0040 (7)	0.0001 (7)
C14	0.0200 (9)	0.0190 (9)	0.0182 (9)	−0.0017 (7)	−0.0048 (7)	0.0001 (7)
C15	0.0228 (10)	0.0177 (9)	0.0214 (9)	−0.0035 (8)	−0.0027 (7)	0.0006 (7)
C16	0.0187 (9)	0.0244 (10)	0.0264 (10)	−0.0046 (8)	−0.0013 (8)	−0.0028 (8)
C17	0.0184 (9)	0.0271 (10)	0.0224 (10)	−0.0019 (8)	−0.0048 (7)	−0.0027 (8)
C18	0.0229 (10)	0.0231 (9)	0.0167 (9)	−0.0008 (8)	−0.0043 (7)	−0.0015 (7)
C19	0.0223 (10)	0.0218 (9)	0.0208 (9)	−0.0066 (8)	−0.0065 (7)	0.0023 (7)
C20	0.0405 (14)	0.0417 (14)	0.0422 (14)	0.0058 (11)	−0.0077 (11)	0.0114 (11)
C21	0.0498 (15)	0.0227 (11)	0.0422 (14)	−0.0134 (10)	−0.0211 (11)	0.0123 (9)
C22	0.0306 (12)	0.0280 (11)	0.0449 (13)	−0.0136 (9)	−0.0218 (10)	0.0096 (10)
C23	0.0269 (10)	0.0263 (10)	0.0252 (10)	−0.0027 (8)	−0.0098 (8)	0.0038 (8)
N5	0.0235 (9)	0.0177 (8)	0.0270 (9)	−0.0053 (7)	−0.0116 (7)	0.0064 (6)

### Geometric parameters (Å, °)

**Table:**

S1—C7	1.738 (2)	C12—H12	0.9500
S1—C9	1.7478 (18)	C13—N5	1.373 (2)
N1—C7	1.356 (2)	C14—C15	1.400 (3)
N1—C6	1.433 (3)	C14—C19	1.402 (3)
N1—C21	1.457 (3)	C14—N5	1.405 (2)
N2—C7	1.313 (2)	C15—C16	1.380 (3)
N2—C8	1.372 (2)	C15—H15	0.9500
N3—C12	1.332 (2)	C16—C17	1.390 (3)
N3—C13	1.351 (2)	C16—H16	0.9500
N4—C13	1.332 (2)	C17—C18	1.388 (3)
N4—C10	1.352 (2)	C17—H17	0.9500
C1—C6	1.367 (3)	C18—C19	1.397 (3)
C1—C2	1.405 (3)	C18—C23	1.509 (3)
C1—C20	1.475 (3)	C19—H19	0.9500
C2—C3	1.349 (4)	C20—H20A	0.9800
C2—H2	0.9500	C20—H20B	0.9800
C3—C4	1.396 (4)	C20—H20C	0.9800
C3—H3	0.9500	C21—H21A	0.9800
C4—C5	1.355 (4)	C21—H21B	0.9800
C4—H4	0.9500	C21—H21C	0.9800
C5—C6	1.432 (4)	C22—H22A	0.9800
C5—H5	0.9500	C22—H22B	0.9800
C8—C9	1.374 (3)	C22—H22C	0.9800
C8—C22	1.499 (3)	C23—H23A	0.9800
C9—C10	1.447 (3)	C23—H23B	0.9800
C10—C11	1.393 (2)	C23—H23C	0.9800
C11—C12	1.380 (3)	N5—H5N	0.8800
C11—H11	0.9500		
			
C7—S1—C9	88.26 (9)	C15—C14—C19	118.35 (17)
C7—N1—C6	120.68 (17)	C15—C14—N5	116.79 (17)
C7—N1—C21	120.59 (17)	C19—C14—N5	124.86 (17)
C6—N1—C21	118.57 (16)	C16—C15—C14	120.77 (18)
C7—N2—C8	109.74 (16)	C16—C15—H15	119.6
C12—N3—C13	114.47 (16)	C14—C15—H15	119.6
C13—N4—C10	117.30 (16)	C15—C16—C17	120.50 (18)
C6—C1—C2	117.4 (2)	C15—C16—H16	119.8
C6—C1—C20	120.8 (2)	C17—C16—H16	119.8
C2—C1—C20	121.8 (2)	C18—C17—C16	119.96 (18)
C3—C2—C1	122.0 (3)	C18—C17—H17	120.0
C3—C2—H2	119.0	C16—C17—H17	120.0
C1—C2—H2	119.0	C17—C18—C19	119.56 (18)
C2—C3—C4	120.4 (2)	C17—C18—C23	121.20 (17)
C2—C3—H3	119.8	C19—C18—C23	119.23 (18)
C4—C3—H3	119.8	C18—C19—C14	120.82 (18)
C5—C4—C3	119.8 (3)	C18—C19—H19	119.6
C5—C4—H4	120.1	C14—C19—H19	119.6
C3—C4—H4	120.1	C1—C20—H20A	109.5
C4—C5—C6	119.3 (2)	C1—C20—H20B	109.5
C4—C5—H5	120.3	H20A—C20—H20B	109.5
C6—C5—H5	120.3	C1—C20—H20C	109.5
C1—C6—C5	121.1 (2)	H20A—C20—H20C	109.5
C1—C6—N1	121.1 (2)	H20B—C20—H20C	109.5
C5—C6—N1	117.8 (2)	N1—C21—H21A	109.5
N2—C7—N1	123.19 (18)	N1—C21—H21B	109.5
N2—C7—S1	116.16 (14)	H21A—C21—H21B	109.5
N1—C7—S1	120.64 (15)	N1—C21—H21C	109.5
N2—C8—C9	116.43 (17)	H21A—C21—H21C	109.5
N2—C8—C22	116.68 (17)	H21B—C21—H21C	109.5
C9—C8—C22	126.83 (18)	C8—C22—H22A	109.5
C8—C9—C10	133.18 (17)	C8—C22—H22B	109.5
C8—C9—S1	109.40 (14)	H22A—C22—H22B	109.5
C10—C9—S1	117.43 (14)	C8—C22—H22C	109.5
N4—C10—C11	120.64 (17)	H22A—C22—H22C	109.5
N4—C10—C9	114.62 (16)	H22B—C22—H22C	109.5
C11—C10—C9	124.73 (17)	C18—C23—H23A	109.5
C12—C11—C10	116.55 (18)	C18—C23—H23B	109.5
C12—C11—H11	121.7	H23A—C23—H23B	109.5
C10—C11—H11	121.7	C18—C23—H23C	109.5
N3—C12—C11	124.37 (17)	H23A—C23—H23C	109.5
N3—C12—H12	117.8	H23B—C23—H23C	109.5
C11—C12—H12	117.8	C13—N5—C14	129.50 (16)
N4—C13—N3	126.49 (17)	C13—N5—H5N	115.3
N4—C13—N5	119.31 (16)	C14—N5—H5N	115.3
N3—C13—N5	114.20 (16)		
			
C6—C1—C2—C3	1.2 (3)	C7—S1—C9—C10	179.10 (16)
C20—C1—C2—C3	−177.0 (2)	C13—N4—C10—C11	−1.6 (3)
C1—C2—C3—C4	−0.5 (4)	C13—N4—C10—C9	179.50 (16)
C2—C3—C4—C5	−0.1 (4)	C8—C9—C10—N4	−176.2 (2)
C3—C4—C5—C6	−0.1 (4)	S1—C9—C10—N4	3.7 (2)
C2—C1—C6—C5	−1.4 (3)	C8—C9—C10—C11	5.0 (4)
C20—C1—C6—C5	176.9 (2)	S1—C9—C10—C11	−175.12 (16)
C2—C1—C6—N1	−179.71 (18)	N4—C10—C11—C12	3.5 (3)
C20—C1—C6—N1	−1.4 (3)	C9—C10—C11—C12	−177.68 (18)
C4—C5—C6—C1	0.9 (4)	C13—N3—C12—C11	−2.2 (3)
C4—C5—C6—N1	179.3 (2)	C10—C11—C12—N3	−1.5 (3)
C7—N1—C6—C1	−79.6 (3)	C10—N4—C13—N3	−2.7 (3)
C21—N1—C6—C1	105.1 (3)	C10—N4—C13—N5	176.82 (18)
C7—N1—C6—C5	102.0 (3)	C12—N3—C13—N4	4.6 (3)
C21—N1—C6—C5	−73.3 (3)	C12—N3—C13—N5	−175.00 (17)
C8—N2—C7—N1	179.19 (19)	C19—C14—C15—C16	1.2 (3)
C8—N2—C7—S1	0.2 (2)	N5—C14—C15—C16	−179.46 (18)
C6—N1—C7—N2	178.0 (2)	C14—C15—C16—C17	−1.6 (3)
C21—N1—C7—N2	−6.9 (3)	C15—C16—C17—C18	0.2 (3)
C6—N1—C7—S1	−3.0 (3)	C16—C17—C18—C19	1.6 (3)
C21—N1—C7—S1	172.13 (18)	C16—C17—C18—C23	−177.98 (18)
C9—S1—C7—N2	0.49 (18)	C17—C18—C19—C14	−1.9 (3)
C9—S1—C7—N1	−178.57 (19)	C23—C18—C19—C14	177.62 (18)
C7—N2—C8—C9	−1.0 (3)	C15—C14—C19—C18	0.6 (3)
C7—N2—C8—C22	176.42 (19)	N5—C14—C19—C18	−178.72 (18)
N2—C8—C9—C10	−178.7 (2)	N4—C13—N5—C14	−14.5 (3)
C22—C8—C9—C10	4.1 (4)	N3—C13—N5—C14	165.06 (19)
N2—C8—C9—S1	1.3 (2)	C15—C14—N5—C13	−160.93 (19)
C22—C8—C9—S1	−175.77 (19)	C19—C14—N5—C13	18.4 (3)
C7—S1—C9—C8	−0.97 (16)		

### Hydrogen-bond geometry (Å, °)

**Table:**

*D*—H···*A*	*D*—H	H···*A*	*D*···*A*	*D*—H···*A*
N5—H5N···N3^i^	0.88	2.22	3.097 (2)	173

Symmetry codes: (i) −*x*+1, −*y*, −*z*+1.
